# Muscle Insulin Resistance and the Inflamed Microvasculature: Fire from Within

**DOI:** 10.3390/ijms20030562

**Published:** 2019-01-29

**Authors:** Jia Liu, Zhenqi Liu

**Affiliations:** Division of Endocrinology and Metabolism, Department of Medicine, University of Virginia Health System, Charlottesville, VA 22908, USA

**Keywords:** insulin resistance, muscle, microvasculature, endothelial cells, inflammation

## Abstract

Insulin is a vascular hormone and regulates vascular tone and reactivity. Muscle is a major insulin target that is responsible for the majority of insulin-stimulated glucose use. Evidence confirms that muscle microvasculature is an important insulin action site and critically regulates insulin delivery to muscle and action on myocytes, thereby affecting insulin-mediated glucose disposal. Insulin via activation of its signaling cascade in the endothelial cells increases muscle microvascular perfusion, which leads to an expansion of the endothelial exchange surface area. Insulin’s microvascular actions closely couple with its metabolic actions in muscle and blockade of insulin-mediated microvascular perfusion reduces insulin-stimulated muscle glucose disposal. Type 2 diabetes is associated with chronic low-grade inflammation, which engenders both metabolic and microvascular insulin resistance through endocrine, autocrine and paracrine actions of multiple pro-inflammatory factors. Here, we review the crucial role of muscle microvasculature in the regulation of insulin action in muscle and how inflammation in the muscle microvasculature affects insulin’s microvascular actions as well as metabolic actions. We propose that microvascular insulin resistance induced by inflammation is an early event in the development of metabolic insulin resistance and eventually type 2 diabetes and its related cardiovascular complications, and thus is a potential therapeutic target for the prevention or treatment of obesity and diabetes.

## 1. Introduction 

Diabetes prevalence continues to increase at an alarming rate and has become the most common and costly chronic disease worldwide. This pandemic currently affects over 400 million individuals, largely due to a rapid increase in the prevalence of type 2 diabetes mellitus (T2DM) [1]. Although the exact mechanisms underpinning the pathogenesis of T2DM remain to be elucidated, the disease is characterized by insulin resistance in multiple organs and systems. Insulin resistance is a complex metabolic abnormality that affects the ability of peripheral tissues to respond to insulin, leading to impaired peripheral tissue glucose utilization and resulting in the development of compensatory hyperinsulinemia and eventually hyperglycemia [2,3]. T2DM confers significant morbidity and mortality to patients by accelerating the development of atherosclerotic complications such as coronary artery disease, cerebrovascular disease and peripheral artery disease and predisposing patients to microvascular complications including retinopathy, nephropathy, and neuropathy [4,5]. Mounting evidence has confirmed that insulin, in addition to stimulating tissue glucose uptake and use [6], is also a vascular hormone that regulates vascular tone and tissue perfusion [7]. In healthy humans, insulin acts on all segments of the arterial tree, induces endothelium-dependent vasorelaxation and increases tissue perfusion [7]. Insulin’s actions in the muscle microvasculature has garnered much attention in the past decade as it is in the muscle microvasculature that the exchanges of nutrients, oxygen, and hormones such as insulin between the plasma compartment and muscle interstitium take place and we and others have convincingly demonstrated that insulin resistance in the muscle microvasculature closely couples with impaired insulin-stimulated glucose use in muscle [8,9,10]. Furthermore, endothelial dysfunction, characterized by abnormal vascular reactivity, increased production of reactive oxygen species (ROS), decreased nitric oxide (NO) bioavailability, and altered barrier function, is increasingly recognized as an early defect that leads to impaired vascular insulin responses and a contributing factor to the pathogenesis of T2DM and its cardiovascular complications [11,12,13]. In this review, we discuss available evidence on the regulation of insulin actions in muscle microvasculature in health and T2DM and the potential role of microvascular inflammation in the development of endothelial and metabolic insulin resistance in muscle.

## 2. Vasculature is a Target for Insulin Action

Insulin, in addition to exerting metabolic actions on traditional peripheral insulin response tissues such as liver, skeletal muscle and adipose tissue, actively regulates vascular tone and tissue perfusion and these actions have been linked to the pathogenesis of diabetes and its cardiovascular complications. Vascular endothelium, a single endothelial cell layer lining the inner surface of the vascular lumen, expresses abundant insulin receptors as well as the insulin-like growth factor I (IGF-1) receptors and the hybrid insulin/IGF-1 receptors [14,15,16,17]. At physiological concentrations, insulin binds and activates the insulin receptors exclusively but, at higher insulin concentrations, as seen in the insulin resistant states or in individuals receiving insulin injections, it also stimulates the IGF-1 receptors and the hybrid insulin/IGF-1 receptors, resulting in the phosphorylation of the endothelial NO synthase (eNOS) at Ser1177 through the phosphoinositide 3-kinase (PI3K)/protein kinase B (PKB or Akt) pathway and the production of NO [14,18,19]. NO is a potent vasodilator as well as an important vascular health keeper as it modulates vascular smooth muscle cell (VSMC) proliferation, reduces the production of adhesion molecules, diminishes endothelial adhesion of inflammatory cells and prevents platelet aggregation to the endothelium. On the other hand, insulin also acts through the mitogen-activated protein kinase (MAPK) pathway to increase the expression and secretion of endothelin-1 (ET-1) by endothelial cells, which binds to the ET-1 receptors to engender vasoconstriction, oxidative stress, and VSMC growth and mitogenesis [20,21,22].

Insulin fine-tunes vascular tone and health via balancing its signals through these two signaling pathways in the basal state but, at high physiological levels, as seen during euglycemic hyperinsulinemic clamp or in the postprandial state, insulin’s vasodilatory effect predominates. Ample evidence has confirmed that insulin exerts a vasoactive action at all levels of the arterial tree. It acts on large conduit arteries to increase compliance [23,24,25], the resistance arterioles to increase overall blood flow to tissue [26,27], the precapillary arterioles to enhance tissue perfusion and the capillaries to expand endothelial exchange surface area and promote substrate exchange between the plasma and tissue interstitium [8,9]. In the insulin resistant state, patients develop compensatory hyperinsulinemia but insulin’s vascular responses through the PI3-kinase/Akt/eNOS pathway are attenuated while its actions via the MAPK pathway remain intact or even enhanced (i.e., a pathway selective insulin resistance) [7,28,29]. As a result, the net (chronic) effects of insulin action on the vasculature are increased production of ET-1 and adhesion molecules and decreased production/bioavailability of NO, tilting the balance towards vasoconstriction and atherosclerosis predisposition [11,30]. Combined, this pathway selective insulin resistance and hyperinsulinemia-driven MAPK signaling likely contribute to the increased vascular tone and heightened predisposition to atherosclerosis and explain the high prevalence of hypertension, tissue hypoxia and macrovascular complications in patients with T2DM.

## 3. Microvasculature Critically Regulates Insulin Action in Muscle

**Figure 1:**
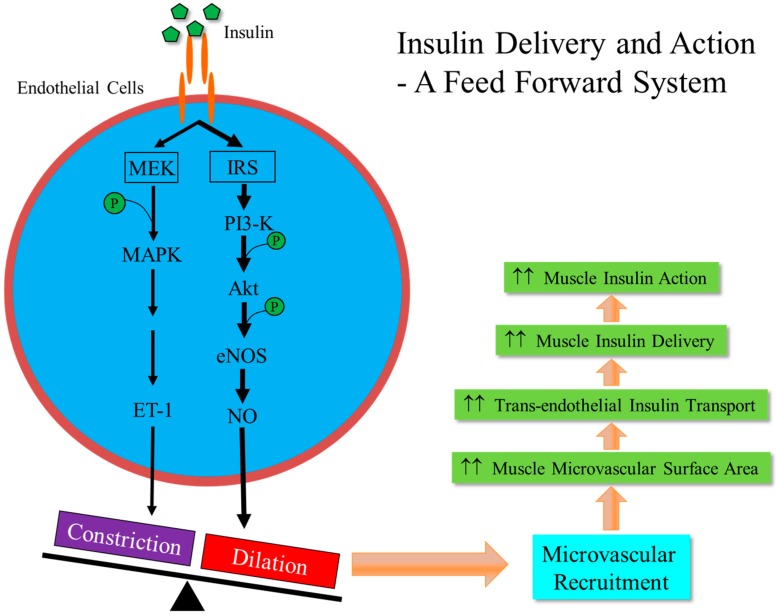
Insulin facilitates its own delivery to muscle through a feed forward system that is rate-limiting in insulin action. In the healthy state, insulin fine-tunes vascular tone and health via balancing its actions through the PI3-K/Akt/eNOS pathway and MAPK/ET-1 pathway. At high physiological levels, insulin’s effects on the PI3-K/Akt/eNOS pathway predominates, causing vasodilation and increased muscle capillary perfusion, a process called microvascular recruitment, and thus insulin delivery to the capillaries nurturing the myocytes. Insulin also enhances its own transport through the endothelial barrier from the plasma compartment to the muscle interstitium. (Abbreviations: IRS, insulin receptor substrates; PI3-K, phosphoinositide 3-kinase; Akt, protein kinase B; eNOS, endothelial NO synthase; MAPK, mitogen-activated protein kinase; ET-1, endothelin-1.)

Skeletal muscle is one of the main targets of insulin and responsible for approximately 80% of insulin-mediated glucose uptake [31,32]. To act on its receptors in the muscle cell membranes to induce subsequent glucose transporter 4 (GLUT4) translocation and glucose uptake [6], insulin has to be first delivered to the capillaries nurturing the muscle fibers and then transported through the endothelial barrier to reach the muscle interstitium. These steps are rate-limiting for insulin action in muscle [33,34,35] and it is the insulin concentrations in the muscle interstitium, not in the plasma, that correlate closely with insulin’s metabolic actions [36]. Insulin itself actively regulates its own delivery to the muscle by increasing muscle perfusion in the microvasculature [37,38] and facilitates its own trans-endothelial transport [39,40] to reach the muscle interstitium prior to its binding to the insulin receptors located in the muscle cell membrane. Muscle microvasculature, including all vessels <150 µm in diameter such as small arterioles, capillaries, and small venules, plays a central role in the regulation of muscle insulin action and substrate metabolism. It provides the needed endothelial surface area for nutrients, oxygen, and hormones such as insulin to enter into the muscle interstitium and for metabolic wastes to be removed from the muscle cells. The skeletal muscle microvasculature behaves in an extremely flexible and reactive manner. In the resting state (i.e., lack of contractile stimulation), approximately only one-third of muscle capillaries are being perfused [41], but, in response to an increased metabolic demand such as during exercise, more capillaries are being perfused via the relaxation of the pre-capillary arterioles, a process termed microvascular recruitment. We and others have confirmed that muscle microvasculature is an important insulin target in that insulin stimulates NO production which causes relaxation of the pre-capillary arterioles to increase muscle microvascular perfusion and expand the exchange surface area. A schema of this process is presented in Figure 1. This insulin-mediated microvascular recruitment occurs rapidly, within 5–10 min, and precedes insulin-stimulated glucose disposal (~20–30 min) in muscle, and inhibition of NO synthesis during insulin infusion abolishes insulin-induced microvascular recruitment in muscle and decreases insulin-stimulated steady-state whole body glucose disposal by up to 40% [37,42]. Thus, insulin’s microvascular and metabolic actions appear to be closely coupled and the metabolic actions of insulin on skeletal muscle depend on the effects of insulin on the skeletal muscle microvasculature as well as the skeletal muscle fibers. The importance of muscle microcirculation in the regulation of insulin’s metabolic actions is further strengthened by many observations in both humans and laboratory animals that multiple factors with proven evidence to affect muscle insulin action and glucose metabolism are able to induce muscle microvasculature recruitment, including exercise [43,44], mixed meals [45,46], resveratrol [47], ranolazine [48], angiotensin II type 1 receptor blockers [49,50,51], angiotensin 1-7 [52], adiponectin [53,54], glucagon-like peptide-1 [55,56], and angiotensin II type 2 receptor agonist [57]. 

The muscle microvascular endothelium layer is continuous without fenestration and has tight junctions between the endothelial cells. The endothelium layer forms a barrier to prevent muscle cells from being exposed to toxic metabolic wastes and harmful inflammatory factors as well as shield the myocytes from being overly stimulated by hormones, nutrients, and numerous other factors, including insulin. Studies using cultured endothelial cells showed that insulin itself also facilitates its own trans-endothelial transport which is dependent on its binding to the insulin receptor and the receptor activation as inhibition of insulin receptor signaling blunts this process [40,58,59]. This in vitro evidence is supported by in vivo study in humans that insulin transport through the vascular endothelium is a saturable and regulated process [39]. Thus, the trans-endothelial transport of insulin appears to be a rate-limiting process for insulin action on muscle. It protects myocytes from insulin overstimulation but also contributes to insulin resistance by slowing down insulin entry into the muscle interstitium, a double-edged sword. Indeed, in skeletal muscle, the delay in insulin-stimulated glucose disposal occurs before insulin receptor binding and is due to the time required for plasma insulin to gain access to the insterstitial compartment [60] and in people with obesity and T2DM, insulin-mediated glucose disposal and muscle glucose uptake are clearly delayed compared to lean individuals [61].

## 4. Metabolic Insulin Resistance Coexists with Microvascular Insulin Resistance

The close coupling between insulin’s microvascular actions and metabolic actions in muscle is further supported by the observation that metabolic insulin resistance coexists with microvascular insulin resistance in both clinical and animal studies. In humans or animals with either chronic or acute (experimental) insulin resistance microvascular responses to insulin are unequivocally reduced [45,54,62,63,64,65,66,67]. Insulin potently recruits muscle microvasculature in healthy humans [68] but fails to do so in obese humans or healthy humans receiving systemic infusion of lipid solution, which acutely raises plasma concentrations of free fatty acids (FFA) and causes metabolic insulin resistance [45,62,63]. Similarly, insulin’s muscle microvascular actions are blunted in animal models of obesity or diabetes [67,69], and the loss of muscle microvascular insulin responses is also apparent in healthy rodents with acute experimental insulin resistance conferred by systemic infusions of factors known to be elevated in the insulin resistant states, such as tumor necrosis factor α (TNF-α) [70] and FFAs [71].

As muscle microvascular endothelial exchange surface area is important in the delivery of insulin to muscle interstitium and thus insulin action in muscle, one would expect that expansion of the microvascular blood volume would result in increased delivery of insulin to the muscle and thus enhance insulin-mediated muscle disposal in the insulin resistant states. Indeed, studies from our and other laboratories have shown repeatedly that expansion of muscle microvascular blood volume with various agents can partially prevent or even reverse insulin resistance in rodents. Administration of losartan acutely expands muscle microvascular blood volume and this effect is associated with increased muscle use of glucose in the post-absorptive state and a full restoration of muscle insulin sensitivity in rats receiving systemic lipid infusion [49,50,51]. Systemic infusion of GLP-1 improves muscle insulin action by potently recruiting muscle microvasculature in rats with either acute (lipid infusion) or chronic (high-fat diet (HFD) feeding) insulin resistance [55,56]. Similarly, muscle contraction has been shown to recruit muscle microvasculature and increase muscle uptake of insulin during a systemic lipid infusion [43,44] and adiponectin, which induces microvascular recruitment via 5’ adenosine monophosphate-activated protein kinase (AMPK) activation and NO production, ameliorates metabolic insulin resistance in rats fed a HFD [53,54]. AMPK can functionally phosphorylate eNOS on multiple sites including Ser633 and Ser1177 to increase endothelial NO production [72,73] Activation of AMPK in addition to regulating NO production also regulates mitochondrial function and substrate metabolism as well as inhibits oxidative stress, ER stress and inflammation [74,75].

Together, these studies highlight an important link between microvascular and metabolic effects of insulin in muscle and indicate that a loss of microvascular insulin responses contributes to the development or worsening of metabolic insulin resistance and expansion of the muscle microvascular volume experimentally enhances metabolic insulin responses. As such, microvascular insulin resistance may play a pathogenesis role in the development of T2DM and a therapeutic target for the prevention and management of T2DM. 

## 5. Metabolic Insulin Resistance is Associated with Inflammation in the Skeletal Muscle

Insulin resistance as seen in obesity and T2DM is associated with a chronic inflammatory state with increased tissue infiltration of immune cells in multiple organs and tissues such as adipose tissue, liver, skeletal muscle, pancreas, and brain, and people with or animal models of obesity and diabetes have elevated plasma levels of inflammatory mediators such as TNF-α, C-reactive protein, and FFAs [2,76,77]. While most studies have focused on inflammation in the adipose tissue, rising evidence suggests that inflammation occurs within skeletal muscle as well, with multiple factors contributing to the pathogenesis of inflammation in muscle, through endocrine, autocrine and paracrine actions. 

First, the circulating pro-inflammatory cytokines secreted and FFAs released from the adipose tissue, particularly the visceral fat depot, have been well studied and confirmed to cause insulin resistance in muscle [78,79,80]. They act through multiple signaling pathways including the NF-κB, JNK and p38 MAPK pathways [81]. In addition, adipose tissue actively secretes a variety of pro- and anti-inflammatory adipokines to modulate insulin action in muscle [82]. In patients with obesity and diabetes, plasma levels of leptin, resistin and visfatin are increased while plasma concentrations of adiponectin is reduced [82]. Second, skeletal muscle itself has been shown to be a secretory organ and myocytes are able to secrete many cytokines, known as myokines, such as interleukin (IL)-6, IL-18, IL-15, irisin, myostatin, and others, that can affect myocytes via autocrine actions and immune cells and microvasculature locally via paracrine actions [83]. The most studied myokine perhaps is IL-6 as its secretion from muscle is markedly increased after exercise and muscle contraction. The effects of IL-6 on insulin sensitivity vary depending on the exposure time. IL-6 acutely increases muscle glucose uptake and fat oxidation, hepatic glucose production, and lipolysis during exercise [84,85] to adapt to increased energy demand from muscle. While acute treatment of myocytes or intravenous infusion of IL-6 to healthy humans increases basal and insulin-stimulated glucose uptake by myocytes and improves whole body insulin sensitivity, chronic action of IL-6 induces insulin resistance [83]. Thus far, there is a clear lack of data on the impact of various myokines on muscle microvascular responses to insulin. Third, muscle infiltration of the immune cells such as macrophages and T lymphocytes, primarily located in the muscle adipose tissue between myocytes or surrounding the muscle (i.e., intermyocellular, intermuscular or perimuscular adipose tissue), that can upon activation secrete inflammatory cytokines [83,86,87]. They act on myocytes and local microvasculature through a paracrine fashion to induce insulin resistance. In addition, an increased influx of FFAs from these ectopic adipose depots to the myocytes and microvasculature within the muscle are certainly adding insults to the insulin signaling pathway and negatively regulate glucose metabolism.

## 6. Inflammation-Induced Insulin Resistance in Muscle Microvasculature is an Early Event in the Development of Obesity and T2DM

Vascular inflammation is a well-recognized phenomenon in obesity and diabetes and contributes to both endothelial dysfunction and insulin resistance, which usually coexist, mutually perpetuate, and contribute to the development of metabolic insulin resistance and related cardiovascular complications. Vascular walls in the insulin resistant state are inflammatory and pro-atherogenic. Serving as a barrier to protect tissue cells with various biological functions from harmful metabolic wastes or products, endothelium is constantly exposed to high levels of circulating inflammatory cytokines, FFAs and insulin, which not only induce insulin resistance in endothelial cells but also significantly increase endothelial cell synthesis of pro-inflammatory factors and adhesion molecules. Insulin at physiological concentrations only selectively stimulates insulin receptors, while it activates insulin as well as IGF-1 signaling in endothelial cells at high concentrations, as seen in the insulin resistant states, and both insulin and IGF-1 receptors mediate insulin stimulated endothelial production of adhesion molecules and monocyte adhesion to the endothelial cells [14]. 

Insulin-mediated microvascular recruitment depends on normal endothelial response to insulin and endothelial dysfunction negatively impacts insulin’s microvascular action thereby contributing to the development of vascular insulin resistance. Inflammation clearly induces microvascular insulin resistance and reduces endothelial NO availability. Pro-inflammatory cytokines increase endothelial production of ROS and cause oxidative stress, leading to increased endothelial generation of superoxide instead of NO, a condition known as uncoupling [88]. Excessive superoxide further reduces NO bioavailability through rapid oxidative inactivation of NO and contributes to endothelial dysfunction and death [88]. Systemic infusion of TNF-α for 3 h completely prevented the insulin-mediated changes in femoral blood flow, vascular resistance, and capillary recruitment, and significantly reduced insulin-stimulated muscle glucose uptake in rodents [70]. Given that endothelial dysfunction and vascular insulin resistance usually coexist and chronic inflammation engenders both, Zhao et al. investigated the temporal relationship between vascular insulin resistance and metabolic insulin resistance and the role of inflammation by assessing insulin responses in all arterial segments, including aorta, distal saphenous artery and the muscle microvasculature, as well as the metabolic insulin responses in rats fed a HFD for various duration ranging from three days to four weeks with or without simultaneous sodium salicylate, an inhibitor of NF-κB [89,90], treatment [65]. HFD feeding induced vascular inflammation and reduced insulin responses in aorta in one week and in small resistance vessel in four weeks but blocked insulin-mediated microvascular recruitment in as early as three days. Insulin-stimulated whole body glucose disposal did not begin to progressively decrease until after one week. Simultaneous treatment with sodium salicylate fully inhibited vascular inflammation, prevented microvascular insulin resistance and significantly improved muscle metabolic responses to insulin. These data are consistent with another report demonstrating that vascular insulin resistance occurs well before muscle insulin resistance in rats fed a HFD [91] and strongly suggest that inflammation-induced microvascular insulin resistance is an early event in diet-induced obesity and this may contribute to the development of metabolic insulin resistance in muscle. It also suggests that muscle microvasculature is a potential therapeutic target for the prevention and treatment of diabetes and its related complications. Indeed, administration of salsalate (a nonacetylated dimer of salicylic acid) for four days to healthy human subjects to inhibit inflammation attenuated FFAs-induced microvascular and metabolic insulin resistance in humans [92]. Treatment of HFD fed rats with liraglutide, a GLP-1 receptor agonist with potent anti-inflammatory property [93,94], for four weeks fully prevented HFD-induced muscle microvascular insulin resistance, restored small arterial vessel endothelial function, and improved insulin-stimulated glucose disposal [64]. 

Inflammation also decreases trans-endothelial insulin transport, a rate limiting step in muscle insulin action. Endothelial cells are able to actively take up insulin which is the first step of the trans-endothelial transport process and this is significantly reduced in the presence of TNF-α [58]. Feeding rats a HFD for as short as one week effectively reduced insulin uptake by (freshly isolated) rat aortic ECs by ~50% [95]. Thus, microvascular inflammation affects both insulin delivery to the microvasculature by blunting insulin-mediated microvascular recruitment and insulin transport to the muscle interstitium by reducing its trans-endothelial transport, two critical steps in the feed-forward insulin delivery system [8,9]. 

In addition to regulating blood flow thus insulin delivery to the muscle, NO also inhibits endothelial cellular senescence [96] and decreased NO availability due to inflammation and oxidative stress may accelerate endothelial senescence and death. Indeed, chronic exposure to TNFα causes premature senescence of endothelial cells, which can be prevented by antioxidant N-actyl cysteine and NF-κB inhibitor plumericin [97]. Insulin at physiological concentrations reduces glucose-induced endothelial senescence, which is associated with reduced ROS and increased NO, and small interfering RNA targeting eNOS reduces the anti-senescence effects of insulin [98]. 

While both the endocrine effects of the circulating cytokines and FFAs and the paracrine effects of myokines from myocytes and cytokines and FFAs from muscle fat depots are important in microvascular inflammation and insulin resistance, the role of perivascular adipose tissue (PVAT), adipose tissues surrounding the small blood vessels, on microvascular function and insulin sensitivity must be emphasized [99]. In recent years, PVAT has been recognized as an important local regulator of vascular function by sensing vascular paracrine signals and secreting a variety of vasoactive adipocytokines [100]. Under physiological conditions, PVAT attenuates agonist-induced vasoconstriction by releasing vasoactive molecules including hydrogen peroxide, angiotensin 1–7, adiponectin, methyl palmitate, hydrogen sulfide, NO and leptin, but this salutary action is lost in the obese state [101]. In small arteries isolated from obese humans, vascular dysfunction is evident but the reduced NO availability and increased ET-1 signaling can be reversed by PVAT removal [102]. PVAT also controls insulin-induced vasoreactivity in the muscle microcirculation through secretion of adiponectin and subsequent AMPKα2 signaling (with resultant eNOS activation and NO production) but obesity-related PVAT inhibits insulin-induced vasodilation, which can be restored by inhibition of the inflammatory kinase JNK in PVAT [103]. The importance of PVAT regulation of muscle microvascular responses to insulin is further supported by a human study demonstrating that, compared with lean women, obese women have impaired insulin-induced microvascular recruitment, lower metabolic insulin sensitivity, and larger muscle perivascular adipocyte size, and PVAT from lean women enhances insulin-induced vasodilation in isolated skeletal muscle resistance arteries while PVAT from obese women leads to insulin-induced vasoconstriction [104]. While the exact explanation to the opposite roles of PVAT in lean *vs.* obese women remains unclear, it is likely that the differences in adipocyte size and cytokine/adipokine profiles may have contributed.

## 7. Conclusions

**Figure 2:**
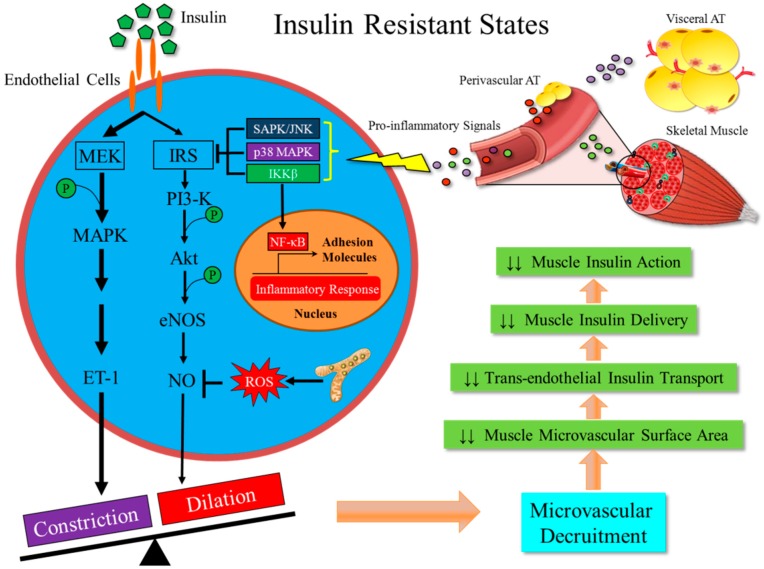
Inflammation in the muscle microvasculature reduces insulin-mediated microvascular recruitment and trans-endothelial insulin transport. In the insulin resistant states, multiple pro-inflammatory factors contribute to the development of inflammation in the muscle microvasculature through a combination of endocrine, paracrine and autocrine actions. Inflammation results in a pathway selective insulin resistance, leading to lower NO bioavailability, less microvascular recruitment and reduced trans-endothelial insulin transport. (Abbreviations: FFAs, free fatty acids; AT, adipose tissue; PVAT, perivascular adipose tissue; NF-κB, nuclear factor kappa-light-chain-enhancer of activated B cells; JNK, c-jun N-terminal kinase). Colored circles are pro-inflammatory factors from various sources.

Insulin is a vasoactive hormone and regulates its own delivery to and action in muscle by relaxing pre-capillary arterioles to recruit microvasculature and facilitating its own trans-endothelial transport. Muscle microvasculature provides the needed endothelial surface area for substrate exchange and insulin transport thereby affecting insulin-mediated glucose disposal and is a key linkage between insulin’s vascular and metabolic actions. Insulin resistance is a low-grade chronic inflammation state and inflammation in the muscle microvasculature blunts insulin-mediated microvascular recruitment and reduces trans-endothelial insulin transport, thus reducing insulin delivery to and action in muscle. A schema of the interplay among muscle microvascular inflammation, insulin delivery and insulin resistance is shown in Figure 2. Currently available data suggest that inflamed endothelium in the muscle microvasculature, i.e., muscle microvascular inflammation, is an early event in obesity-induced insulin resistance and a pivotal node linking microvascular and metabolic insulin resistance. Thus, early interventions aiming at preventing endothelial dysfunction and ameliorating inflammation in muscle microvasculature may help diabetes prevention and control.
